# Peanut Sprout Extracts Attenuate Triglyceride Accumulation by Promoting Mitochondrial Fatty Acid Oxidation in Adipocytes

**DOI:** 10.3390/ijms20051216

**Published:** 2019-03-11

**Authors:** Seok Hee Seo, Sang-Mi Jo, Jiyoung Kim, Myoungsook Lee, Yunkyoung Lee, Inhae Kang

**Affiliations:** 1 Department of Marine Life Science, Jeju National University, Jeju 63243, Korea; bossni3@naver.com; 2 Department of Food Science and Nutrition, Jeju National University, Jeju 63243, Korea; iris1603@hanmail.net (S.-M.J.); lyk1230@jejunu.ac.kr (Y.L.); 3 Department of Food and Nutrition, Kyungnam College of Information & Technology, Pusan 47011, Korea; kjy1@eagle.kit.ac.kr; 4 Department of Food and Nutrition, Sungshin Women’s University, Seoul 01133, Korea; mlee@sungshin.ac.kr

**Keywords:** peanut sprouts, resveratrol, adipogenesis, fatty acid oxidation, mitochondrial respiration

## Abstract

Peanut sprouts (PS), which are germinated peanut seeds, have recently been reported to have anti-oxidant, anti-inflammatory, and anti-obesity effects. However, the underlying mechanisms by which PS modulates lipid metabolism are largely unknown. To address this question, serial doses of PS extract (PSE) were added to 3T3-L1 cells during adipocyte differentiation. PSE (25 µg/mL) significantly attenuated adipogenesis by inhibiting lipid accumulation in addition to reducing the level of adipogenic protein and gene expression with the activation of AMP-activated protein kinase (AMPK). Other adipocyte cell models such as mouse embryonic fibroblasts C3H10T1/2 and primary adipocytes also confirmed the anti-adipogenic properties of PSE. Next, we investigated whether PSE attenuated lipid accumulation in mature adipocytes. We found that PSE significantly suppressed lipogenic gene expression, while fatty acid (FA) oxidation genes were upregulated. Augmentation of FA oxidation by PSE in mature 3T3-L1 adipocytes was confirmed via a radiolabeled-FA oxidation rate experiment by measuring the conversion of [^3^H]-oleic acid (OA) to [^3^H]-H_2_O. Furthermore, PSE enhanced the mitochondrial oxygen consumption rate (OCR), especially maximal respiration, and beige adipocyte formation in adipocytes. In summary, PSE was effective in reducing lipid accumulation in 3T3-L1 adipocytes through mitochondrial fatty acid oxidation involved in AMPK and mitochondrial activation.

## 1. Introduction

The prevalence of obesity has reached epidemic proportions in the United States and all over the world [1,2]. Obesity is characterized by the abnormal expansion of white adipose tissue, either due to an increase in the number of adipocytes from pre-adipocytes or an increase in cell size [3,4]. Obesity increases the risk of metabolic disorders, such as type 2 diabetes, heart disease, hypertension, and cancer [5]. Therefore, understanding the molecular mechanisms by which hyperplastic and hypertrophic obesity is driven by adipocytes and identifying the novel molecules for regulating lipid metabolism is necessary for the prevention of obesity. 

The peanut (*Arachis hypogaea* L.) is a naturally occurring, common, nutritious food, which contains high levels of protein, unsaturated fatty acid, fiber, potassium, magnesium, copper niacin, arginine, fiber, α-tocopherol, and folates. Bioactive compounds, such as phytosterols, and flavonoids are also rich in the peanut [6]. Peanut seeds can be germinated to create a form called peanut sprouts (PS). PS have a high total polyphenolic content, including a high content of resveratrol, protocatechuic acid, gallic acid, and caffeic acid [7]. Among them, resveratrol (3,5,4′-*trihydroxystilbene*) is a well-known major component of PS. It has been previously reported that the amount of resveratrol significantly increases from 4.5 µg/g on day 0 of germination to around 30 µg/g on day 9 of germination [7]. The health-promoting effects of resveratrol are mainly due to the activation of AMP-activated protein kinase (AMPK)/sirtuin 1 (SIRT1), which regulates energy metabolism in the whole body [8,9]. In particular, resveratrol supplementations have been shown to increase mitochondrial content/activity in skeletal muscle, brown adipose tissue, and the liver. This enhances body basal energy expenditure, which protects against diet-induced obesity and metabolic complications such as insulin resistance and fatty liver disease [8]. Accumulating evidence has reported that PS has anti-oxidant [10], anti-inflammatory [11], and lipid-lowering effects in vivo [12,13] and in vitro [14]. However, most studies were descriptive reports and few studies have explored the role of PS in lipid accumulation and its effect on adipocytes. 

In the present study, we first report that PS extract (PSE) is a potent negative regulator of adipogenesis in 3T3-L1 cells adipocytes. We also confirm the anti-adipogenic properties of PSE by using another adipocyte model that does not need clonal expansion such as C3H10T1/2 and primary adipocytes from ear mesenchymal stem cells (EMSCs) [15]. To investigate the regulatory effects of PSE on lipid metabolism, 3T3-L1 adipocytes were used to examine the induction of fatty acid oxidation, energy metabolism-related genes and mitochondrial bioenergetics.

## 2. Results

### 2.1. Total Polyphenol, Flavonoid, and Resveratrol Contents of PS Extracts

The total polyphenol content of the PSE was 10.87 mg gallic acid/g extract, while the total flavonoid content was 3.79 mg catechin/g extract (Table 1). The resveratrol content of PSE, determined by LC/MS, was 18 μg/g. 

### 2.2. Effects of PSE on Cell Viability 3T3-L1 and C3H10T1/2 Preadipocytes

**Figure 1:**
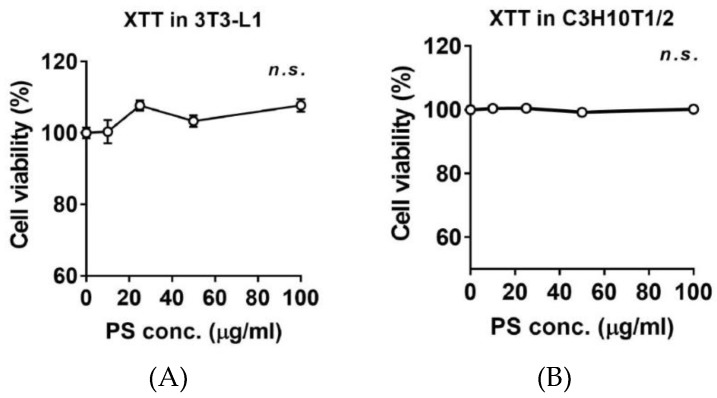
Effects of PSE on cell viability in 3T3-L1 and C3H10T1/2 pre-adipocytes. The culture of 3T3-L1 (**A**) and C3H1051/2 cells (**B**) were treated with 10‒100 μg/mL of PSE for 24 h. XTT reagent was added 3 h before measurement of OD 450 nm. Data are expressed as a percentage of the vehicle control (dimethyl sulfoxide (DMSO)). *n.s.* represents no significance. Data are represented as the mean ± SEM of three independent experiments. Values that do not share the same superscript are significantly different, as determined by one-way ANOVA (*p* < 0.05).

To determine whether PSE affects the cell viability of pre-adipocytes, the cytotoxic effects of PSE (10–100 μg/mL) were determined in both 3T3-L1 and C3H10T1/2 pre-adipocytes. PSE was incubated for 24 h before the 2,3-Bis-(2-methoxy-4-nitro-5-sulfophenyl)-2H-tetrazolium-5-carboxanilide salt (XTT) assay. As shown in Figure 1, there was no significant reduction in cell viability after different doses of PSE were applied. 

### 2.3. PSE Inhibits Adipogenesis in 3T3-L1 Adipocytes

**Figure 2:**
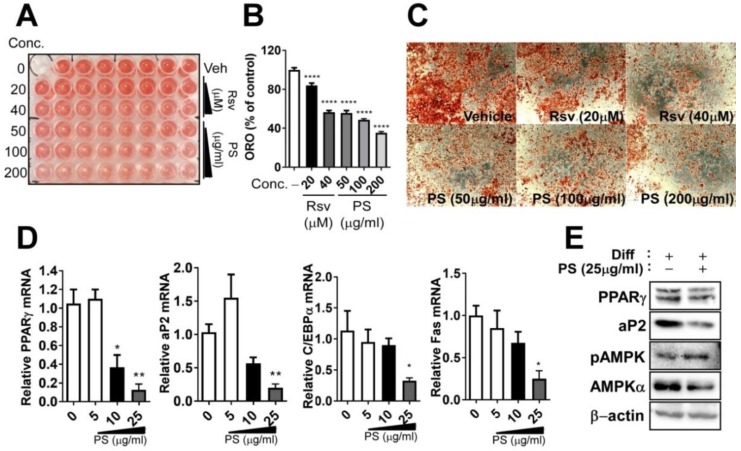
PSE inhibits adipogenesis in 3T3-L1 adipocytes. 3T3-L1 cells were seeded and induced to differentiation in the presence of DMSO (vehicle control), resveratrol (20–40 μM) or PSE (50–200 μg/mL) for 10 days: (**A**) TG accumulation in 96-well culture plates was visualized by ORO staining; (**B**) extracted ORO staining was quantified (OD 500 nm); (**C**) representative images from three separate experiments (magnified 4×); (**D**) adipogenic gene expression of PPARγ, aP2, C/EBP α, and Fas by qPCR; (**E**) adipogenic protein expressions of PPARγ, aP2, phosphor-specific, or total antibodies targeting AMPK and β-actin by Western blot analysis. All values are presented as the mean ±S.E.M. * *p* < 0.05; ** *p* < 0.01; *** *p* < 0.001; **** *p* < 0.0001 compared with the vehicle control (DMSO treated cells) by one-way ANOVA with Bonferroni’s comparison test. +; treatment, -; non-treatment.

To determine whether PSE was able to inhibit adipogenesis, PSE was added to 3T3-L1 cells during differentiation; this was maintained for 10 days. The presence (50, 100, 200 μg/mL) of PSE caused a significant reduction in triglyceride (TG) accumulation, as measured by Oil-red-O (ORO) staining (Figure 2A–C), which is compatible with 40 μM of resveratrol treatment (resveratrol was used as a positive control). Next, we investigated whether a low dose of PSE (5–25 μg/mL) has anti-adipogenic effects. Adipogenic gene and protein expressions were determined by quantitative PCR (qPCR) and Western blot. PSE treatment, especially 25 μg/mL, significantly suppressed adipogenic gene expression, including peroxisome proliferator-activated receptor gamma (PPARγ), fatty acid binding protein 4 (FABP4, aP2), CCAAT/enhancer binding protein α (C/EBPα), and fatty acid synthase (Fas) (Figure 2D). Based on these results, we used the 25 μg/mL concentration of PSE for the rest of the experiments to ensure we did not cause cellular damage. Adipogenic protein expression, including PPARγ and aP2, was also reduced in murine cultures treated with 25 μg/mL of PSE (Figure 2E). 

AMP kinase (AMPK) activation is a well-known mechanism behind the anti-adipogenic properties of several phytochemicals [16,17,18,19]. In our study, treatment with PSE (25 μg/mL) increased AMPK phosphorylation (Figure 2E).

### 2.4. PSE Inhibits Adipogenesis in C3H10T1/2 Mouse Embryonic Fibroblasts Adipocytes and EMSCs

**Figure 3:**
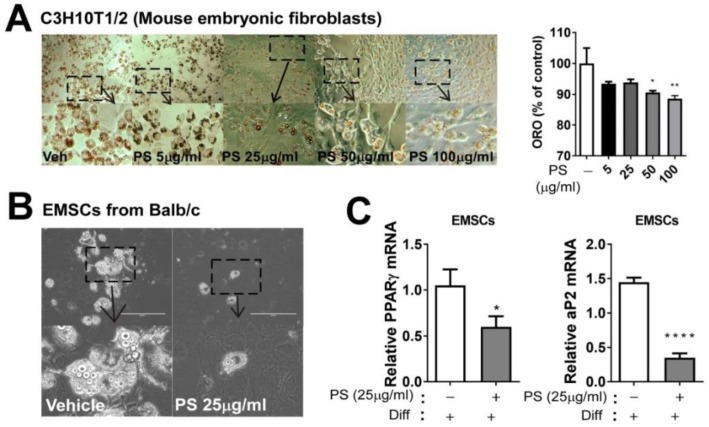
PSE inhibits adipogenesis in C3H10T1/2 mouse embryonic fibroblasts, adipocytes, and EMSCs. C3H10T1/2 cells were seeded and induced to differentiation in the presence of either DMSO (vehicle control) and PSE (5–100 μg/mL) for four days: (**A**) Triglyceride accumulation was visualized by Oil red-O staining and representative images from three separate experiments are shown in (**left**) (magnified 4×); extracted ORO staining was quantified (OD 500 nm) (**right**). Primary adipocytes were prepared from EMSC of Balb/c mice: (**B**) Phase contrast images of primary adipocytes were differentiated with or without PSE (25 μg/mL) for seven days (magnified 4×, scale bar = 400 μm); (**C**) adipogenic gene expression of PPARγ and aP2 by qPCR. * *p* < 0.05; ** *p* < 0.01; **** *p* < 0.0001 compared with the vehicle control (DMSO treated cells) by one-way ANOVA with Bonferroni’s comparison test or Student’s t-test. +; treatment, -; non-treatment.

We also utilized a complementary approach by using the models do not required clonal expansion to confirm the anti-adipogenic effects of PSE, which were C3H10T1/2 mouse embryonic fibroblasts mimicking primary mouse embryo-fibroblasts [15] and mouse primary adipocytes from EMSCs. PSE (50 and 100 μg/mL) caused a significant reduction in TG accumulation, as measured by ORO staining in C3H10T1/2 cells (Figure 3A). To further confirm the anti-adipogenic effects of PSE, we prepared primary adipocytes derived from EMSCs. The bright-field picture represents the differentiated primary adipocytes and PSE (25 μg/mL) significantly inhibited lipid droplet formation (Figure 3B). Consistent with reduced TG accumulation, the adipogenic gene PPARγ, aP2, C/EBP α, and Fas expression were significantly suppressed by PSE (Figure 3C). 

### 2.5. PSE Attenuates Lipid Accumulation in Cultures of Adipocytes by Upregulating Fatty Acid Oxidation and Mitochondrial Oxygen Consumption

**Figure 4:**
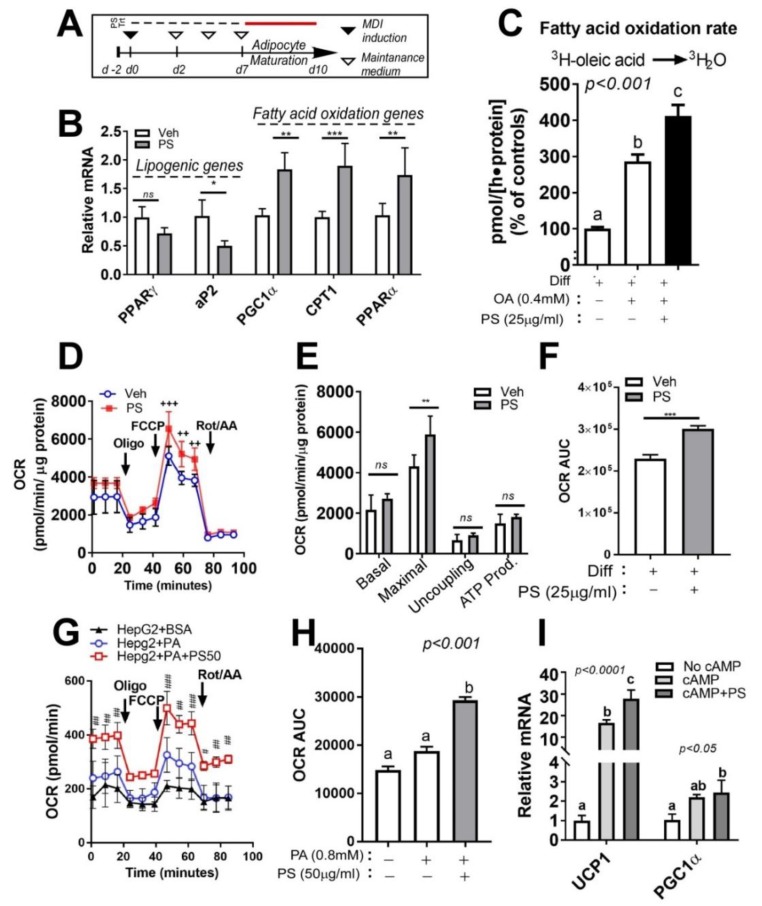
PSE attenuates lipid accumulation in cultures of adipocytes by upregulating fatty acid oxidation and mitochondrial oxygen consumption. (**A**) Experimental scheme. 3T3-L1 were seeded on the second day before differentiation (*d-2*) and induced to differentiation (*d0*, MDI: methyl isobutyl- xanthine, dexamethasone, and insulin). Keep 3T3-L1 cells differentiated into fully differentiated adipocytes until d7. Fully differentiated adipocytes (*d7*) were incubated with PSE (25 μg/mL) for three days. (**B**) Lipogenic and fatty acid oxidation-related gene expression of PPARγ, aP2, PGC1α, CPT1, and PPARα as determined by qPCR. (**C**) Conversion of [^3^H]-OA into [^3^H]-H_2_O. (**D**–**F**) Oxygen consumption rate (OCR) in 3T3-L1 adipocytes treated with Veh (blue) and PSE (red) as determined by Seahorse extracellular analyzer. 3T3-L1 cells differentiated into fully differentiated adipocytes. Fully differentiated adipocytes (*d7*) were incubated with PSE (25 μg/mL) for one day. Arrow indicates the addition of respiratory inhibitors of oligomycin (Oligo), carbonyl cyanide 4-trifluoromethoxy phenylhydrazone (FCCP) and a combination of antimycin A and rotenone (Rot/AA). (**G**–**H**) OCR in HepG2 cells treated with BSA (black), PA (blue), and PA + PSE (red) as determined by Seahorse extracellular analyzer. HepG2 cells were pre-incubated with PSE (50 μg/mL)for 48 h. BSA or 0.8 mM BSA-PA complex was loaded for 3 h. (I) Relative expressions of UCP1 and PGC1α by qPCR. Pre-treatment of the 3T3-L1 cell with PSE for 7 d during adipogenesis, followed by Bt2-cAMP stimulation for 6 h. All values are presented as the mean ±SEM. *n.s.* represents no significance. * *p* < 0.05; ** *p* < 0.01; *** *p* < 0.001 compared with the vehicle control (DMSO-treated cells) by Student’s *t*-test or one-way ANOVA with Bonferroni’s comparison test. Means that do not share a common superscript are significantly different as determined by one-way ANOVA with Bonferroni’s comparison test. ++ *p* < 0.01; +++ *p* < 0.001 compared with the vehicle control (DMSO treated cells) # *p* < 0.05; ## *p* < 0.01; ### *p* < 0.001 compared with PA-treated HepG2 cells by two-way ANOVA with Bonferroni’s comparison test. +; treatment, -; non-treatment.

We next postulated that PSE would antagonize adipocyte hypertrophy. To address this hypothesis, fully differentiated cultures of 3T3-L1 adipocytes were exposed to PSE (25 μg/mL) for three days based on the experimental design (Figure 4A). Exposure to PSE (25 μg/mL) caused a significant reduction of lipogenic activation as measured by mRNA expression while there was upregulation of fatty acid oxidation-related gene expression (peroxisome proliferator-activated receptor gamma coactivator 1-alpha (PGC1α), carnitine palmitoyltransferase I (CPT1), PPARα) (Figure 4B). 

Next, we investigated the enhancement of FA oxidation in PSE-treated cells by determination of the actual FA oxidation rate using a radiolabeled precursor. [^3^H]-OA was added to fully differentiated adipocytes for 2 h, then the conversion of [^3^H]-H_2_O released from [^3^H]-OA was measured by a liquid scintillation counter. BSA-OA complex treatment significantly increased the FA oxidation rate, which confirmed the accuracy of this method. PSE (25 μg/mL) markedly increased the conversion of [^3^H]-OA to [^3^H]-H_2_O (Figure 4C). 

To determine the impact of PSE on mitochondrial activities, OCR was determined by the Seahorse system, which is a standard method for exhibiting the key parameters of the mitochondrial function. PSE significantly increased maximal respiration compared to the vehicle control. Moreover, we also found an increase in the area under the curve (AUC) of OCR (Figure 4D–F). Mitochondrial bioenergetics properties of PSE were also confirmed in hepatoma HepG2 cells by measuring OCR. Palmitic acid (PA) treatment conjugated with BSA has a marginal increase of OCR compared to the BSA-treated group. Pretreatment 50 μg/mL of PSE enhanced OCR even more than PA-treated HepG2 cells (Figure 4G,H). 

After this, we investigated whether the upregulation of FA oxidation and alterations in white adipocyte mitochondrial respiratory function of PSE are involved in the augmentation of beige adipocyte formation. Differentiated 3T3-L1 adipocytes were treated with dibutyryl cyclic AMP (Bt2-cAMP), an analog of cAMP, for mimicking beige adipocytes in the presence or absence of PSE (25 μg/mL). The incubation of mature adipocytes with Bt2-cAMP was correlated with the induction of uncoupling protein 1 (UCP1) gene expression, which confirmed the accuracy of this analog. Interestingly, PSE significantly enhanced UCP1 levels at an even higher level compared to Bt2-cAMP-treated adipocytes. PSE also significantly enhanced the PGC1α gene expression (Figure 4I).

## 3. Discussion

Peanuts (*Arachis hydrogaea* L.) have nutritional characteristics that can promote human health [6,20,21,22]. Recent reports have suggested that peanut sprouts prepared from the germination of peanut kernels are rich in phytochemicals. It is suggested that resveratrol (3,5,4’-*trihydroxystilbene*), which is a naturally occurring polyphenol present in grapes, berries, and other vegetables, is the major phytochemical in peanut sprouts. Resveratrol is synthesized by several plants as a defense mechanism against stress, such as UV irradiation and/or microbial infection [23,24]. In rodents, resveratrol improves mitochondrial and metabolic health by modulating energy metabolism via activation of the AMPK/Sirtuin 1 (SIRT1)/PGC-1α axis [8,9]. Some clinical studies have confirmed the metabolic effects of resveratrol [25,26]. Since several recent reports showed the beneficial health effects of PSE, such as anti-inflammatory, anti-oxidant, and anti-obesity effects [10,11,12,13], it is plausible to assume that PSE may regulate AMPK/SIRT activation in a similar way to resveratrol to control lipid metabolism in adipocytes. However, the effect of PSE on lipid metabolism through stimulating changes in adipocytes and uncovering its detailed molecular mechanisms have not been sufficiently studied. Here, we demonstrate that PSE is effective at attenuating TG accumulation and increasing mitochondrial FA oxidation involved in AMPK mechanisms, which may ultimately modulate energy metabolism. These results may provide insight into PSE as a unique therapeutic method for controlling adiposity. 

In this study, we noticed that PSE from PS that germinated for nine days had a high total polyphenols content (TPC) and total flavonoid content (TFC); moreover, the resveratrol contents were consistent with other reports [7] (Table 1), which indicates that PS may be a potentially functionally beneficial food. Therefore, we decided that it is appropriate to investigate the lipid modulating properties of PSE in adipocytes. By using the high potency of PSE, which has high TPC, TFC, and resveratrol contents, we first assessed the cytotoxicity of PSE in 3T3-L1 and C3H10T1/2 pre-adipocyte cells. Consistently, the accumulating data suggested that PSE treatment does not seem to affect cell viability in several non-carcinogenic cells [27,28]. In a study by Kim et al. [14], there was no significant difference in the cell cytotoxicity of 3T3-L1 cells within two days of PS ethanol treatment (up to 40 μg/mL). In our data, up to 100 µg/mL concentrations of PSE showed no sign of cytotoxicity in the tested time frames for both 3T3-L1 and C3H10T1/2 cells (Figure 1). Based on ORO data, adipogenic gene and protein data, and a literature review of routine PSE concentrations for cellular studies (25–100 µg/mL), we chose the 25 µg/mL concentration of PSE as a non-toxic concentration to assess the potency of PSE in regulating lipid metabolism in adipocytes.

In our study, PSE treatment in adipocytes was associated with at least three metabolic consequences: (i) inhibition of adipogenesis in 3T3-L1 adipocytes and other adipocyte models, (ii) enhancement of fatty acid oxidation and mitochondrial respiration, which was correlated to AMPK activation, and (iii) increase in beige adipogenic conversion pathways of white adipocytes. 

The first metabolic outcome that we immediately noticed was that PSE impacted adipogenesis in several adipocyte cell models (3T3-L1, C3H10T1/2 cells and primary adipocytes from EMSCs) (Figure 1 and Figure 2). In our data, neutral TG accumulation was reduced significantly from 50–200 µg/mL, which is similar to 40 µM of resveratrol treatment (Figure 1A–C). We wondered whether the lower concentration of PSE was also able to inhibit adipogenic and protein expression during adipogenesis and 25 µg/mL of PSE concentration effectively inhibited adipogenic conversion in 3T3-L1 cells. In agreement with our notion, Kim et al. reported that PS ethanol extract (40 µg/mL) significantly attenuated adipogenesis in 3T3-L1 cells [14]. We extracted PSE components with the pressurized hot water extraction method, not ethanol extraction. It is unclear what concentrations and/or types of bioactive components are created by PS extraction (pressurized hot water extraction vs. ethanol extraction), so it would be interesting to compare these two methods regarding the biochemical and functional aspects of PSE. One of the possible mechanisms that involve inhibiting adipogenesis of PSE is the inhibition of matrix metalloproteinases (MMP), which are differentially expressed in adipose tissue during obesity and modulate adipocyte differentiation [29]. Extensive extracellular matrix occurs during adipose tissue growth and MMP, especially MMP2 and MMP9, which play a pivotal role in regulating adipose tissue remodeling. Interestingly, a recent report showed that PSE attenuates MMP2 and MMP9 activity during adipogenesis in 3T3-L1 adipocytes [14]. Apart from PSE, several other functional food components, such as ginseng [30], have been found to regulate MMP during adipogenesis. Further studies are necessary to validate the aforementioned MMP inhibition activity by PSE in adipogenic differentiation. In vivo experiments confirmed the lipid-lowering effects of PSE by using high-fat-diet-fed Sprague–Dawley rats [12,13]. Although we confirmed the in vitro anti-adipogenic properties of PSE in our study, we next need to confirm whether it is translated into an animal and/or human.

Subsequently, we examined the anti-lipogenic properties of PSE by using mature adipocytes. PSE directly acts on adipocytes and triggers changes in gene expression and related biochemical parameters, which are consistent with reduced lipogenesis and enhanced substrate oxidation (Figure 4B). This is confirmed by the FA oxidation rate using a radiolabeled precursor (Figure 4C) and maximal OCR (Figure 4D–F). Since AMPK is a major energy sensor that triggers a variety of catabolic processes and suppresses anabolic pathways simultaneously, it is plausible to connect the lipid-lowering effects of PSE and increase mitochondrial fatty acid oxidation with AMPK activation (Figure 2E). Until now, little information has been available regarding the AMPK activation by PSE, although a considerable number of reports have shown an increase in AMPK/SIRT1 due to resveratrol. Resveratrol activates the AMPK/SIRT1 axis, which inhibits adipogenesis and enhances brown adipocyte formation in vivo and in vitro [9]. Resveratrol 20 µM is required to inhibit adipogenesis ([19,31] and Figure 2A–C), and our PS extract contains 18 µg/g resveratrol (Table 1). Although 18 µg/g resveratrol is a significant amount in PSE, it is possible to assume that the upregulation of mitochondrial FA oxidation and AMPK activation by PSE was not solely due to resveratrol. We should examine whether the beneficial effects of PSE in our study were due to resveratrol or the synergistic effects of other bioactive components. Several plant-derived nutrients, such as Salvianolic acid B [32], anthocyanins [33], and ecliptal, a natural compound isolated from the herb *Eclipta alba*, were recently reported to promote mitochondrial respirations in 3T3-L1 adipocytes. In our study, maximal respiration from OCR, which is regarded as an index of energetic reserve capacity, increased after PSE treatment. This indicated that PSE has the potential to have mitochondrial bioenergetic properties. Although HepG2 cells require a higher concentration of PSE (50 µg/mL) than adipocytes, OCR was also upregulated in a non-adipocyte model in our study (Figure 4G–H). We guess that the low sensitivity of PSE was due to the characteristics of HepG2 cells, which are hepatocyte carcinoma cells, not primary hepatocytes [34]. We are currently undertaking a dose- and time-dependent experiment using HepG2 cells to unravel these issues. Since the liver and adipose tissue are the two major organs regulating systemic lipid metabolism, there needs to be an investigation into the lipid-lowering properties of PSE in the liver. Gomez-Zorita et al. also showed that resveratrol-treated obese Zucker rats had reduced fat accumulation and increased fatty acid oxidation in the liver [35]. Our data showed possible mitochondrial bioenergetic properties in the liver, but it is still unclear whether PSE has the potential to modulate hepatic steatosis. We are currently undertaking several experiments related to the hepatic lipid modulation of PSE by mitochondrial activations. 

Finally, we have found the impact of PSE on the beigeing of white adipocytes. It has been suggested that resveratrol could trigger an enhancement in mitochondrial function and metabolic homeostasis for beigeing and brown adipogenesis [31]. In agreement with this notion, our data showed that PSE treatment increased the level of Bt2-cAMP-mediated UCP1 expression to higher than the expression triggered by Bt2-cAMP treatment alone (Figure 4I). This is also involved in the induction of maximal respiration confirmed by seahorse oxygen consumption rate (Figure 4D–F). Since 3T3-L1 cells are a classical white adipocyte model, not a ‘good’ beige adipocyte model, we are currently conducting mechanistic experiments on PSE for the beigeing of white adipocytes in other brite adipocyte models and also examining the protective effects of PSE on *Lipopolysaccharides* (LPS), which are known as lipoglycans/endotoxin-mediated downregulation of UCP1 levels. Further studies are warranted to investigate these possibilities by measuring metabolic activities in the presence or absence of PSE. 

In conclusion, the present study determined that PSE impeded lipid accumulation in adipocytes by the augmentation of mitochondrial fatty acid oxidation, which may mimic resveratrol. A major limitation of our study is that it is still vague whether a 25 µg/mL PSE concentration is feasible in the plasma or adipose depots after nutritional intervention. Thus, determining the clinical relevance and efficacy of PSE supplementation should be conducted with caution. We are currently planning to perform in vivo experiments to confirm PSE’s lipid-modulating effects. Nevertheless, we believe that these discoveries support the potential benefit of PSE as a novel anti-adipogenic and anti-lipogenic agent in future clinical studies.

## 4. Materials and Methods

### 4.1. Materials

All cell cultures were purchased from SPL (Seoul, Korea). Fetal bovine serum (FBS) and penicillin-streptomycin were purchased from Cellgro Mediatech, Inc. (Herndon, VA, USA). Rosiglitazone (BRL49653) was purchased from Cayman Chemical (Ann Arbor, MI, USA). All other chemicals and reagents were purchased from Sigma Chemical Co (St. Louis, MO, USA) unless otherwise stated.

### 4.2. Sample Preparation

PS, germinated for nine days, were kindly provided by WooYoung E&T (Jeju, South Korea). PS were dried, freeze-dried, powdered, and extracted using the pressurized hot water extraction method (modified from [36]). A 10-g sample (dry weight) of PS was mixed with 100 mL of Milli-Q water. The extracted solutions were combined and centrifuged at 3000 rpm for 3 min. Next, the obtained extracts were filtered using Whatman® filter paper and the filtrate was lyophilized to obtain the powdered extract. Finally, the obtained sample was dissolved in dimethyl sulfoxide (DMSO, Sigma, St. Louis, MO, USA) at a concentration of 100 mg/mL with several aliquots and utilized freshly in the in vitro experiments.

### 4.3. Total Polyphenol and Flavonoid Contents of PS Extracts

The total polyphenols content (TPC) of the PS extract was determined using the modified Folin–Denis method [37]. The same volume of PS extracts (50 μL) and 1 M Folin–Ciocalteu’s phenol reagent (FMD Millipore Corporation, Darmstadt, Germany) was added to each well of a 96-well plate and left at room temperature for 5 min. A total of 100 μL of 4% Na_2_CO_3_ solution was added to the reaction, which was incubated for another 60 min at room temperature and protected from light using aluminum foil. The absorbance was obtained at 725 nm at room temperature by a spectrophotometer (Molecular Devices, San Jose, CA, USA) and the results were expressed as gallic acid concentration equivalents.

The total flavonoid content (TFC) was determined by the method of Moreno et al. [38]. We mixed 0.1 mL of the sample with 500 μL of water before 30 μL of a solution containing 5% sodium nitrate was added and the mixture was incubated for 6 min at room temperature. A total of 60 μL of aluminum nitrate (10%, *w*/*v*) was added and the mixture was incubated at 25 °C for 5 min. A total of 60 μL of sodium hydroxide was added and the reactant absorbance was measured at 510 nm. The calibration curve was calculated using catechin and the results were expressed in mg of catechin equivalent.

### 4.4. LC/MS Analyses of PS Extracts

Resveratrol content was determined by LC/MS. Extracts analyzed by HPLC tandem mass spectrometry (LC-MS) were prepared in 0.1% formic acid. A Shimadzu LC system (Kyoto, Japan) was equipped with a Poroshell 120 EC-C18 (3.0 × 50 mm) column. The column temperature was fixed at 40 °C. Mobile phases were 0.1% formic acid in water (solvent A) and 0.1% formic acid in Acetonitrile (solvent B). The gradient was 0 min (60% A), 5 min (30% A), 8 min (5% A), 10 min (5% A), 10.01 min (60% A), and 15 min (60% A). This was followed by 0 min (40% B), 5 min (70% B), 8 min (95% B), 10 min (95% B), 10.01 min (40% B), and 15 min (40% B). The column effluent was monitored at 280 nm and mass spectra data were acquired by electrospray ionization (ESI) in the positive ion mode with a Tandem Mass Spectrometry(API 3200). The source temperature was 500 °C. LC-MS data were collected and processed by Analyst 1.6.2. (SCIEX) (Concord, Ontario, Canada).

### 4.5. Cell Culture

The 3T3-L1 cells (American Type Culture Collection (ATCC), Manassas, VA, USA) were grown to confluence in a basal medium—Dulbecco’s modified Eagle’s medium (Sigma) with 50 IU/mL penicillin (Sigma), 50 μg/mL streptomycin (Sigma) and 2 mM l-glutamine (Sigma)—supplemented with 10% newborn calf serum (Linus, Madrid, Spain). Two days after the cells reached confluence (referred as Day 0), they were induced to differentiate in a basal medium containing 10% fetal bovine serum (FBS; Invitrogen, Carlsbad, CA, USA), 1 μM dexamethasone (DEX; Sigma), 0.5 mM methylisobutylxanthine (MIX; Sigma) and 1 μg/mL insulin (Sigma) for 48 h. This was followed by 48 h in a basal medium containing 10% FBS and 1 μg/mL insulin. The cells were subsequently refed fresh basal medium supplemented with 10% FBS (without insulin) every other day. For browning induction of white adipocytes, cells were treated with dibutyryl cyclic adenosine monophosphate (cAMP) (Bt2-cAMP; 0.5 mM) for 6 h at the end of adipocyte differentiation.

C3H10T1/2 cells were obtained from the ATCC and maintained in DMEM supplemented with 2 mM L-glutamine, 100 units/mL penicillin, 100 g/mL streptomycin and 10% (*v*/*v*) heat-inactivated fetal bovine serum in a humidified 5% CO_2_ atmosphere at 37 °C. To induce adipocyte differentiation, cells (1 × 10^6^ cells/mL) were grown to 70–80% confluency. Differentiation was induced 2 days later by adding 10 μM of rosiglitazone in an adipogenesis-inducing medium containing 1 µM dexamethasone, 0.5 mM isobutyl-methylxantine, 0.01 mg/mL insulin and 10% FBS in DMEM. After 72 h, the medium was changed every other day to an adipogenesis-inducing medium containing insulin (0.01 mg/mL) and 1 μM of rosiglitazone. 

To prepare the primary cultures of rodent adipocytes, ear mesenchymal stem cells (EMSC) were obtained from the ears of Balb/c mice. Briefly, EMSC were isolated from the pools of 6–8 ears from adult mice (*n* = 3–4) by collagenase digestion (2 mg/mL). The confluent cultures of EMSC were stimulated with an adipogenic differentiation mixture according to standard adipocyte differentiation protocols [39]. 

HepG2 cells were kindly provided by Dr. Shin and cells were originally obtained from the Korean Cell Line Bank (KCLB, Seoul, South Korea). HepG2 cells were maintained in Dulbecco’s modification of Eagle’s medium (DMEM)/Ham’s F12 containing 1 mM glucose, 1% L-glutamine, 10% fetal bovine serum, 100 units/mL penicillin, and 100 g/mL streptomycin in 5% CO_2_ at 37 °C and used for OCR.

### 4.6. Cell Viability Assay

The cytotoxic effect of PSE was determined using the XTT cell viability kit (Cell Signaling Technology, Beverly, MA, USA) according to the manufacturer’s protocol. Briefly, 3T3-L1 and C3H10T1/2 cells were cultured in 96-well plates with a seeding density of approximately 20,000 cells/well. Cells were incubated with either DMSO or increasing concentrations of PSE for 24 h (Figure 1). After this, the medium was replaced with a fresh medium containing XTT solution for 3 h at 37 °C before measurement of OD 450 nm using a microplate reader.

### 4.7. Lipid Accumulation

To measure the lipid accumulation in adipocytes, cells were fixed with 10% formalin and stained with oil red O (ORO). Bright-field images were taken by CKX41 Inverted Microscope (Olympus, Melville, NY, USA) and ORO dye was extracted by isopropanol to quantify the relative triglyceride (TG) accumulation (at OD 500 nm).

### 4.8. Total RNA Extraction and qPCR

Gene-specific primers for qPCR were obtained from Cosmo Genetech (Seoul, Korea). Total RNA was isolated with Trizol reagent (Invitrogen). To remove potential genomic DNA contamination, mRNA was treated with DNase (Mediatech). RNA concentrations were measured by the NanoDrop (Nano-200 Micro-Spectrophotometer, Hangzhou City, China). A total of 1 μg of mRNA was converted into cDNA in a total volume of 20 μL (High-capacity cDNA reverse transcription kits, Applied Biosystems, Foster City, CA, USA). Gene expression was determined by real-time qPCR (CFX96™ Real-Time PCR Detection System, Bio-Rad, CA, USA) and relative gene expression was normalized by hypoxanthine guanine phosphoribosyltransferase (HPRT) and/or glyceraldehyde 3-phosphate dehydrogenase (GAPDH) (primer sequences are available in Table S1).

### 4.9. Western Blot Analysis

To prepare total cell lysates, monolayers of 3T3-L1 adipocytes were scraped with ice-cold radioimmune precipitation assay (RIPA) buffer (Thermo Scientific, Waltham, MA, USA) containing protease inhibitors (Sigma). Proteins were fractionated using 8% or 10% SDS-PAGE, transferred to PVDF membranes and incubated with the relevant antibodies. Chemiluminescence from the ECL (Western Lightning) solution was detected with ChemiDoc (Bio-Rad, Hercules, CA, USA). Polyclonal or monoclonal antibodies targeting phospho-AMPK (Thr172, #2535), total AMPK (#5831), PPARγ (#2435), and β-actin (#4967) were purchased from Cell Signaling Technology. The mouse monoclonal antibodies for aP2 (sc-271529) were purchased from Santa Cruz Biotechnology (Santa Cruz, CA, USA).

### 4.10. Fatty Oxidation Rate Using [^3^H]-OA

To measure the FA oxidation rate, we followed the previously published methods by Olpin et al. and Kang et al. [17,40], who utilized cultures of mature 3T3-L1 adipocytes. Briefly, cells were incubated with serum-free low glucose (1000 mg/L d-(+)-glucose) overnight before the experiment. [^3^H]-OA (Perkin Elmer, Norwalk, CT, USA; final concentration of 0.5 μCi/mL) were mixed with a sodium oleate–bovine serum albumin (BSA) complex (400 μM), before this was added to cells and the mixture incubated for 2 h. The [^3^H] radioactive containing medium was harvested and precipitated using 100% trichloroacetic acid (TCA) solution. After precipitation, we added 6N NaOH to reach a final concentration of 0.8–1.0 N to obtain an alkaline supernatant. The supernatant was run through columns filled with Dowex ion-exchange resin (Acros Organics, AC202971000, Geel, Belgium) to capture [^3^H]-H_2_O. Radioactivity was measured by MicroBeta Microplate counters (PerkinElmer, (Norwalk, CT, USA).

### 4.11. Oxygen Consumption Rate (OCR) by Seahorse

To determine the mitochondrial respiration activities, the O_2_ concentration in the 3T3-L1 adipocytes and human hepatoma HepG2 cells were measured using a XF24 extracellular flux analyzer (Agilent Technologies, Santa Clara, CA, USA). Briefly, 3T3-L1 cells were seeded in a gelatin-coated seahorse microplate (24-well) until they reached confluence, which was followed by adipogenic differentiation as described above. HepG2 cells were pre-incubated with PSE (50 μg/mL) or DMSO for 48 h and 0.8 mM BSA-palmitic acid (PA) complex was loaded for 3 h. The mitochondrial basal respiration was assessed in untreated cells. The cells were then treated with oligomycin (oligo, 2 μM) to measure the ATP turnover. The maximum respiratory capacity was assessed by the addition of carbonyl cyanide 4-trifluoromethoxy phenylhydrazone (FCCP, 0.5 μM), which is a chemical uncoupler of electron transport and oxidative phosphorylation. The mitochondrial respiration was blocked by a combination of antimycin A (1 μM) and rotenone (1 μM) (A + R). The OCR was calculated by plotting the O_2_ tension of the medium in the microenvironment above the cells as a function of time, which was normalized by protein concentrations and expressed in pmol O_2_/min/μg protein.

### 4.12. Statistical Analysis

All the data were expressed as means ± standard error (SE), and statistical calculations were performed using the *t*-test and ANOVA (one-way analysis of variance) with Tukey’s and Bonferroni’s multiple comparison tests. Results were considered significant if *p* < 0.05 (GraphPad Prism Version 7.0, La Jolla, CA, USA).

## Figures and Tables

**Table 1:** Total polyphenol, flavonoid, and resveratrol contents of peanut sprout extract.

Total polyphenols, Flavonoids, and Resveratrol Contents	Peanut Sprout Extract
Total polyphenols (mg Gallic acid/extract g)	10.87 ± 0.11
Total flavonoids (mg Catechin/extract g)	3.79 ± 0.14 ^1^
Resveratrol (μg/g)	18

^1^ Values are presented as the mean ± SEM of three independent experiments.

## Supplementary Materials

Supplementary materials can be found at https://www.mdpi.com/1422-0067/20/5/1216/s1.

## Abbreviations

AMPK	AMP-activated protein kinase
aP2, FABP4	fatty acid binding protein 4
AUC	area under the curve
cAMP	Cyclic adenosine monophosphate
CPT1	Carnitine palmitoyltransferase I
EMSCs	Ear mesenchymal stem cells
FA	fatty acid
MDI	methylisobutylxanthine, dexamethasone, and insulin
OA	oleic acid
OCR	oxygen consumption rate
ORO	oil red O
PA	Palmitic acid
PGC1α	Peroxisome proliferator-activated receptor gamma coactivator 1-alpha
PPARγ	Peroxisome proliferator-activated receptor gamma
PS	Peanut sprouts
PSE	Peanut sprout extracts
SIRT1	sirtuin 1
TFC	total flavonoid contents
TPC	total polyphenol contents
UCP1	uncoupling protein 1
