# Supplementary material for: Peanut Sprout Extracts Attenuate Triglyceride Accumulation by Promoting Mitochondrial Fatty Acid Oxidation in Adipocytes
Source: Int J Mol Sci. 2019 Mar 11;20(5):1216. doi: 10.3390/ijms20051216 (PMC6429123; doi:10.3390/ijms20051216)
Supplement: Supplementary file 1 [file ijms-20-01216-s001.pdf]

Table S 1. Primer sequences for qPCR

Gene	Forward/Reverse	Sequence (5′-3′)
mPPARγ	Forward	GGCGATCTTGACAGGAAAGAC
	Reverse	CCCTTGAAAAATTCGGATGG
maP2	Forward	AGCATCATAACCCTAGATGGCG
	Reverse	CATAACACATTCCACCACCAGC
mC/EBPα	Forward	GGTTTTGCTCTGATTCTTGCC
	Reverse	CGAAAAAACCCAAACATCCC
mFas	Forward	GGAGGTGGTGATAGCCGGTAT
	Reverse	TGGGTAATCCATAGAGCCCAG
mPPARα	Forward	ACGATGCTGTCCTCCTTGATG
	Reverse	GTGTGATAAAGCCATTGCCGT
mCPT1	Forward	CCAGGCTACAGTGGGACATT
	Reverse	AAGGAATGCAGGTCCACATC
mPGC1α	Forward	CCCTGCCATTGTTAAGACC
	Reverse	TGCTGCTGTTCCTGTTTTC
mGAPDH	Forward	CATGGCCTTCCGTGTTCCTA
	Reverse	GCGGCACGTCAGATCCA
mHPRT	Forward	TTGCTCGAGATGTCATGAAGGA
	Reverse	AGCAGGTCAGCAAAGAACTTATAGC
